# Self-Enhanced Near-Infrared Copper Nanoscale Electrochemiluminescence Probe for the Sensitive Detection of Ciprofloxacin in Foods

**DOI:** 10.3390/foods14030538

**Published:** 2025-02-06

**Authors:** Jie Wu, Yuanjie Qin, Xiaoxin Mei, Lin Cai, Wen Hao, Guozhen Fang

**Affiliations:** State Key Laboratory of Food Nutrition and Safety, Tianjin University of Science and Technology, Tianjin 300457, China; 22845807@mail.tust.edu.cn (J.W.); qinyuanjie@mail.tust.edu.cn (Y.Q.); meixiaoxin@mail.tust.edu.cn (X.M.); cailin@mail.tust.edu.cn (L.C.); haowen@mail.tust.edu.cn (W.H.)

**Keywords:** near-infrared electrochemiluminescence, molecularly imprinted polymer, polyvinylpyrrolidone, copper nanowires, ciprofloxacin

## Abstract

Ciprofloxacin (CIP), a widely used broad-spectrum antibiotic, poses a serious threat to human health and environmental safety due to its residues. The complementary monomers molecularly imprinted electrochemiluminescence sensor (MIECLS) based on a polyvinylpyrrolidone-functionalized copper nanowires (CuNWs@PVP) luminescent probe was constructed for the ultra-sensitive detection of CIP. CuNWs with low cost and high conductivity exhibited near-infrared electrochemiluminescence (NIR ECL) properties, yet their self-aggregation and oxidation led to a weakened emission phenomenon. PVP with solvent affinity and large skeleton was in situ attached to CuNWs surface to avoid CuNWs sedimentation and aggregation, and self-enhanced ECL signals were achieved. The bifunctional monomers molecularly imprinted polymer (MIP) possessed complementary active centers that increased their affinity with CIP, enhancing the accurate and sensitive detection of the target substances. The linear range of CIP using MIECLS was 5.00 × 10^−9^–5.00 × 10^−5^ mol L^−1^ with a low limit of detection (LOD) of 2.59 × 10^−9^ mol L^−1^, while the recovery rates of CIP in the spiking recovery experiment were 84.39% to 92.48%. The combination of bifunctional monomer MIP and NIR copper-based nano-luminescent probe provides a new method for the detection of CIP in food.

## 1. Introduction

Ciprofloxacin (CIP), a synthetic drug belonging to the third generation of fluoroquinolone antibiotics, is widely used in the anti-infective treatment of pigs and poultry due to its broad-spectrum and highly effective bactericidal properties [1]. However, it is noteworthy that the conversion rate of CIPs in organisms is less than 30% [2], a large amount of unmetabolized CIP may enter the aquatic environment and foodstuffs through the food chain, posing a potential threat to human health and environmental safety. To address this challenge, several CIP detection methods have been reported, including high-performance liquid chromatography [3], liquid chromatography–mass spectrometry [4], and Raman spectroscopy [5], which provide powerful tools for the effective monitoring of CIP residues. However, these detection methods suffer from drawbacks such as complexity, high costs, and potential interferences [2], and ongoing efforts to develop simpler, more cost-effective, and robust detection techniques remain crucial.

Electrochemiluminescence (ECL) is a phenomenon of light emission induced by electrochemical excitation at electrode surfaces. It has emerged as a powerful analytical technique, distinguished by its remarkable sensitivity, extensive detection range, high level of controllability, and straightforward operational procedures [6]. Within this field, the design of ECL luminophores constitutes the topic at the forefront of investigation. Compared to visible light luminophores, near-infrared electrochemiluminescence (NIR ECL) luminophores with strong tissue penetration ability and low chemical damage to reduce the disintegration of the structure of target substances [7]. However, the potential toxicity of numerous luminophores limits their further application in practical scenarios [8]. Consequently, the exploration and development of NIR ECL luminophores that possess both low toxicity and high luminescent efficiency are regarded as a pivotal breakthrough in the realm of non-interfering sensing and detection.

Copper-based nanomaterials are notable for their abundant resources, low cost, and similar electrical conductivity to silver [9]. Among them, one-dimensional copper nanowires (CuNWs) have shown great potential for exploration in the field of ECL sensing due to their large aspect ratio, low resistance, and excellent transparency [10]. However, the high mass and low surface charge content of CuNWs in solution often cause them to tend to sedimentation and self-aggregation, impairing their optical and electrical properties [11]. To prepare homogeneous and stable configurations of CuNWs, surface functionalization becomes an effective strategy to be considered, including both covalent and non-covalent functionalization. Although effective, covalent functionalization is usually performed under acidic conditions, which may adversely affect the structure and intrinsic properties of CuNWs. In contrast, non-covalent functionalization can achieve good dispersion while preserving the inherent properties of the material by introducing dispersants [12]. By forming a stable coating on CuNWs, which prevents direct CuNW-to-CuNW contact to enhance their dispersion stability in water and organic solvents. A pivotal challenge in non-covalent functionalization lies in selecting appropriate dispersants that can effectively engage with CuNWs while preserving their essential optical and electrical properties.

Polyvinylpyrrolidone (PVP), a nonionic polymer, has a wide range of applications in nanomaterial dispersion, surface activation, and reducing agents [11]. Recent studies have shown that PVP was able to interact with noble metal nanoparticles via the nitrogen atoms and carbonyl groups of the pyrrole ring, enhancing the dispersion of nanomaterials through van der Waals forces [13]. Furthermore, the hydrophobic carbon chains (methylene and methyl groups) in the main chain of PVP exhibited mutual repulsion and spatial site-blocking effects, effectively mitigating agglomeration among nanomaterials [14]. Therefore, CuNWs with non-covalent functionalization using PVP are expected to exhibit excellent dispersion and ECL properties. In addition, the CuNWs aqueous solution with high surface atomic exposure has higher reactivity, leading to a decrease in their long-term stability [9]. Fortunately, organic solvent systems (ethanol) can provide oxidative protection for CuNWs and extend their lifetime [15].

The precise detection of CIP in complex matrices necessitates the highly specific recognition elements, which possess the capability to unequivocally identify and bind to the target molecules. The molecularly imprinted polymer (MIP) employs the principle of molecular recognition, in which the spatial size and three-dimensional structure of target molecules are “imprinted” into a polymer membrane via specific coordination or chemical reactions, thereby facilitating the specific detection of the target molecules [16]. As the core component of MIPs, functional monomers interact with template molecules, forming the basis of MIPs’ molecular recognition ability, and their selection and design directly dictate the selectivity, affinity, and binding ability of MIPs towards target substances [17]. Conventional MIPs possesses only a single functional monomer, leading to potential insufficient affinity and restricted target recognition, owing to their homogeneous structure and single functional group type [18]. In comparison, bifunctional monomers have complementary recognition cavities and are anticipated to exhibit superior selectivity and responsiveness. The emergence of molecularly imprinted sensors (MIECLS) with bifunctional monomers is essential to improve detection efficiency and safeguard product quality and safety, infusing renewed vigor into the innovation and advancement of pesticide and veterinary drug residue detection technologies.

**Scheme 1:**
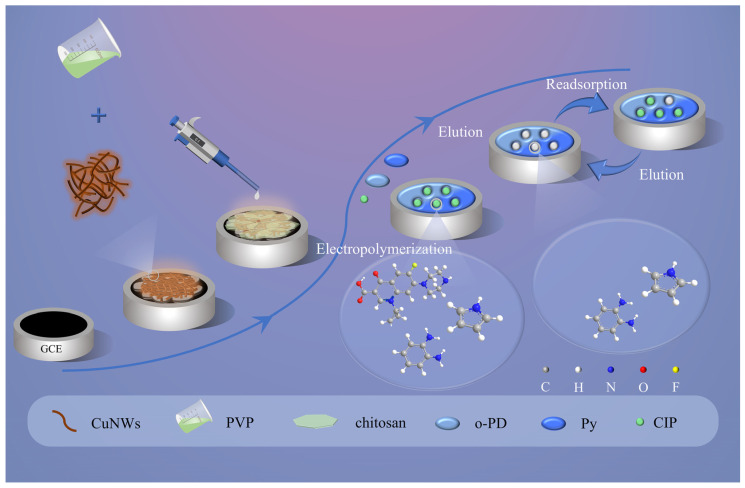
The design principle of MIP/CuNWs@PVP/GCE and the specific detection mechanism for CIP.

Based on the above analysis, the self-enhanced NIR ECL probe CuNWs@PVP, in conjunction with MIP possessing bifunctional monomers, had achieved specificity detection of CIP. As illustrated in Scheme 1, the one-dimensional CuNWs with high transparency and excellent electron transport capability cross-arranged to form an optoelectronic network that exhibited NIR ECL properties. A protective PVP film was adsorbed onto the CuNWs surface, where the solvent dispersion and steric hindrance of the macromolecular PVP, synergically prevented CuNWs from self-aggregating and realized the NIR ECL in situ self-enhancement of CuNWs. The transfer of CuNWs from the traditional water system to the ethanol system resulted in a reduction in the reaction rate with oxygen, further enhancing the stability of the optical signals. Additionally, the meticulously designed bifunctional monomers MIP not only strengthened the recognition ability towards CIP but also extenuated the collapse of the MIP network structure induced by the repeated elution. This work advances the exploration of efficient NIR ECL probes in the fields of environmental monitoring, bioanalysis, clinical medicine, and light-emitting devices and provides new ideas for the specific detection of CIP in complex matrices.

## 2. Materials and Methods

### 2.1. Reagents

CuNWs were acquired from XFNANO (Nanjing, China). CIP, norfloxacin (NOR), enoxacin (ENX), lomefloxacin (LMF), and ofloxacin (OFX) were sourced from Aladdin Science and Technology Co., Ltd. (Shanghai, China). Additionally, *o*-phenylenediamine (*o*-PD), pyrrole (Py), acetic acid, ethanol, methanol, acetonitrile, n-hexane, potassium persulfate, chitosan, phosphate buffer, phosphoric acid, triethylamine, ultra-pure water, alumina powders, SPE column were supplied by Macklin Science and Technology Ltd. (Shanghai, China). Nitrogen was sourced from Tianjin Junliangcheng Gas Co., Ltd. (Tianjin, China). The glass carbon electrode was provided by Tianjin Gaoshi Ruilian Technology Co., Ltd. (Tianjin, China). The infrared lamp was supplied by Royal Philips Electronics N.V. (Eindhoven, The Netherlands), and the refrigerator was provided by Thermo Fisher Scientific Inc. (Waltham, MA, USA).

### 2.2. Synthesis of CuNWs@PVP

The CuNWs water dispersion was centrifuged at 7000 rpm for 6 min, and the collected precipitates were redissolved in deoxidized ethanol solution (blown with nitrogen for 10 min to remove oxygen) to centrifugate for 6 min, and the CuNWs powder was obtained through vacuum drying at 60 °C. We weighed 2 mg PVP and 20 mg CuNWs, and added them into 20 mL deoxy ethanol and stirred at room temperature for 20 min to prepare the CuNWs@PVP composite, which was stored in a refrigerator at 4 °C away from light for use.

### 2.3. Fabrication Process of CuNWs@PVP/GCE

The glassy carbon electrode (GCE) underwent polishing with 0.3 µm and 0.05 µm alumina powders and was rinsed sequentially with ultra-pure water and ethanol to ensure thorough cleaning. Subsequently, the 10 μL of CuNWs@PVP ethanolic dispersion and 4 μL of 0.1% chitosan solution were carefully dropped onto the surface of the dried GCE, which was rotated under an infrared lamp to facilitate the uniform evaporation of the solution to obtain the modified electrode CuNWs@PVP/GCE.

### 2.4. Preparation of MIP/CuNWs@PVP/GCE and NIPCuNWs@PVP/GCE

The prepared CuNWs@PVP/GCE was placed in an acetic acid buffer solution (pH = 5.2, blown with nitrogen for 10 min to remove oxygen) containing 1 mM CIP, 5 mM *o*-PD, and 2 mM Py, and was electropolymerized for 10 cycles within a voltage range of 0 V to 0.8 V at a scan rate of 50 mV s^–1^ to obtain the precursor of MIP/CuNWs@PVP/GCE, and a methanol/acetic acid = 6:1 (*v*/*v*) mixture, eluted for 9 min, was utilized to obtain MIP/CuNWs@PVP/GCE. The NIP/CuNWs@PVP/GCE was prepared using the same method but without the participation of CIP.

### 2.5. Testing Conditions and Instruments

The ECL test was conducted on an MPI-EII ECL analyzer (Xian Remix Co., LTD., Xian, China) in a PBS (pH = 7.4) solution containing 0.1 M K_2_S_2_O_8_ with a photomultiplier tube with an operating voltage of 750 V and a scanning potential ranging from −1.8 V to −0.2 V. The cyclic voltammetry currents (CV) were performed on a CHI660E electrochemical workstation (Shanghai Chenhua Instrument Co., Ltd., Shanghai, China) at a scan rate of 50 mV s^−1^ within a voltage range of 0 V to 0.8 V. The electrochemical impedance spectroscopy (EIS) measurements were carried out on a PARSTAT2273 electrochemical workstation (Ametek Co., Ltd., Berwyn, PA, USA) under the conditions of a 0.0 V direct current voltage, a 10.0 mV alternating current voltage, and a frequency range of 100 MHz to 100 kHz. The testing environment was a 5 mM [Fe (CN) _6_]^3−^/^4−^ solution. Fourier transform infrared spectrometer (FTIR) analysis was conducted utilizing an IS50 Fourier Infrared Spectrometer (Thermo Fisher Scientific Inc., Waltham, MA, USA). The scanning electron microscope (SEM) experiments were conducted utilizing a JSM-IT300 field emission scanning electron microscope manufactured by JEOL Corporation (Tokyo, Japan). For transmission electron microscope (TEM) analysis, a Jem-2100F transmission electron microscope from JEOL Corporation (Tokyo, Japan) was employed.

### 2.6. Real Sample Preparation

The pork and fish samples were sourced from local supermarkets located in Tianjin. Following the Chinese Ministry of Agriculture Bulletin No. 1025-14-2008 [19], the pre-treatment procedure of real samples involved the following steps: after passing through the meat grinder, 2 g of meat samples were weighed in a 30 mL centrifuge tube, followed by the addition of 10 mL of phosphate buffer, swirled for 5 min, and then centrifuged at 1000 rpm for 10 min. This step was repeated twice to obtain the supernatant. For further purification of the sample, it was processed using a solid-phase extraction (SPE) column that was activated with 2 mL of methanol, and subsequently with 2 mL of phosphate buffer. In addition, 5 mL of the prepared supernatant was loaded onto the SPE column, which was rinsed with 1 mL of ultra-pure water to eliminate residual impurities, and 1 mL of a solution consisting of 0.05 M phosphoric acid/triethylamine and acetonitrile 82:18 (*v*/*v*) eluted the target substances, and the resultant eluate was collected for subsequent MIECLS analysis.

## 3. Results

### 3.1. Morphology Characterization of MIP/CuNWs@PVP/GCE

**Figure 1:**
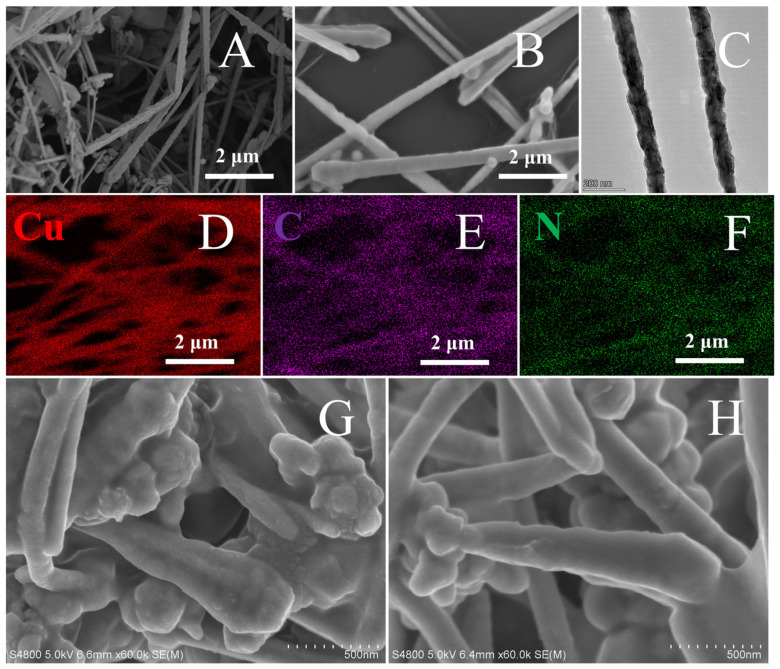
The SEM images of CuNWs (**A**) and CuNWs@PVP (**B**); the TEM image of CuNWs@PVP (**C**); the SEM element mapping of CuNWs@PVP ((**D**–**F**), red signifies Cu, purple signifies C, and green signifies N); the SEM images before (**G**) and after (**H**) elution of MIP/CuNWs@PVP/GCE.

SEM is able to capture high-resolution images of the material surface, while TEM, with its unique advantages, deeply reveals the internal structure of materials, both playing crucial roles in delving into microstructure characteristics. As illustrated in Figure 1A (SEM), CuNWs showed a nanowire aggregation state structure. When PVP adhered to the surface of CuNWs as a functionalized surfactant (Figure 1B, SEM and Figure 1C, TEM), CuNWs were effectively prevented from self-aggregation. CuNWs@PVP was uniform in size and cross-arranged with each other to form a hierarchical interwoven network structure, which was a self-enhanced NIR ECL probe with luminescent and conductive bifunctional properties. Meanwhile, the SEM element mapping by energy-dispersive X-ray spectroscopy (Figure 1D–F) clearly showed the uniform distribution of Cu, C, and N elements, which proved the successful synthesis of the self-enhanced functionalized luminescent probe CuNWs@PVP. The surface of CuNWs@PVP/GCE became roughened following the electropolymerization of a dense MIP membrane consisting of template molecules and functional monomers (Figure 1G). As shown in Figure 1H, during the elution, the force between the template molecules and the functional monomers was disrupted, allowing the template molecules to be removed, and the surface of MIP/CuNWs@PVP/GCE became loose and porous, further demonstrating the successful formation of imprinted pores specifically recognizing CIP.

### 3.2. NIR ECL and Fourier Transform Infrared Spectroscopy Characterization

**Figure 2:**
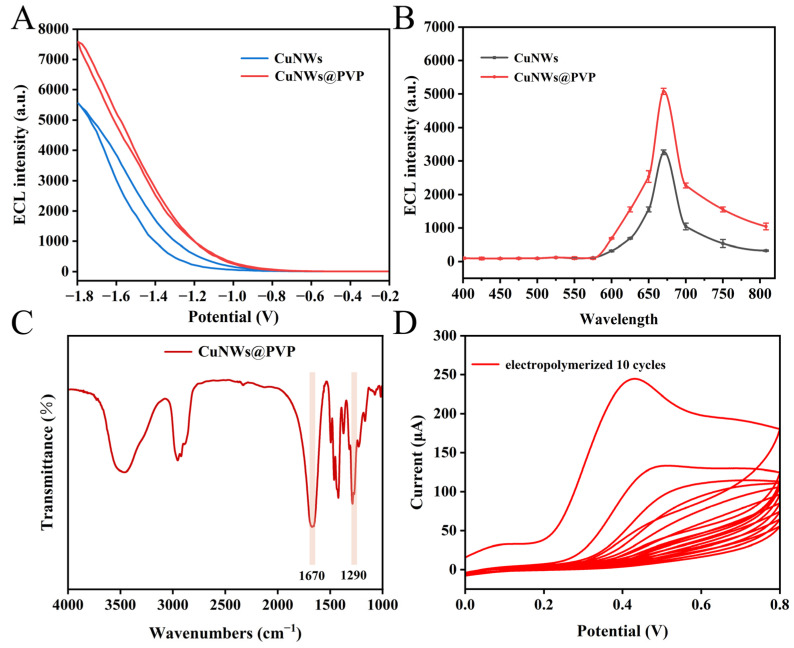
ECL spectrum with voltage variation (**A**); ECL intensity under different wavelength filters (**B**); the Fourier transform infrared spectrum of CuNWs@PVP (**C**); MIP electropolymerization for 10 cycles (**D**).

The measurement of ECL wavelength holds a pivotal role in gaining insights into the properties of probes, providing important reference for the research of materials science, energy science, and other fields. The maximum emission wavelength of CuNWs@PVP was evaluated using ECL filters with various wavelengths ranging from 400 to 808 nm, within a voltage span of −1.8 V to −0.2 V (Figure 2A). Remarkably, the ECL emission peaked at 670 nm (Figure 2B), demonstrating the NIR ECL properties of the CuNWs@PVP, which gives them advantages of strong tissue penetration, little photochemical damage, high stability, and low background interference during actual detection.

To verify the successful synthesis and chemical structure of CuNWs@PVP, FTIR spectroscopy was employed for in-depth analysis. As illustrated in Figure 2C, the prominent absorption peak located around 1670 cm^−1^ corresponded to the stretching vibration of the C=O bond, while the peak near 1290 cm^−1^ was attributed to the stretching vibration of the C-N bond, which was consistent with the literature report [20], proving that the surfactant PVP was successfully attached to CuNWs to facilitate CuNWs@PVP’s durable and stable NIR ECL signals.

### 3.3. Characterization of MIP/CuNWs@PVP/GCE

**Figure 3:**
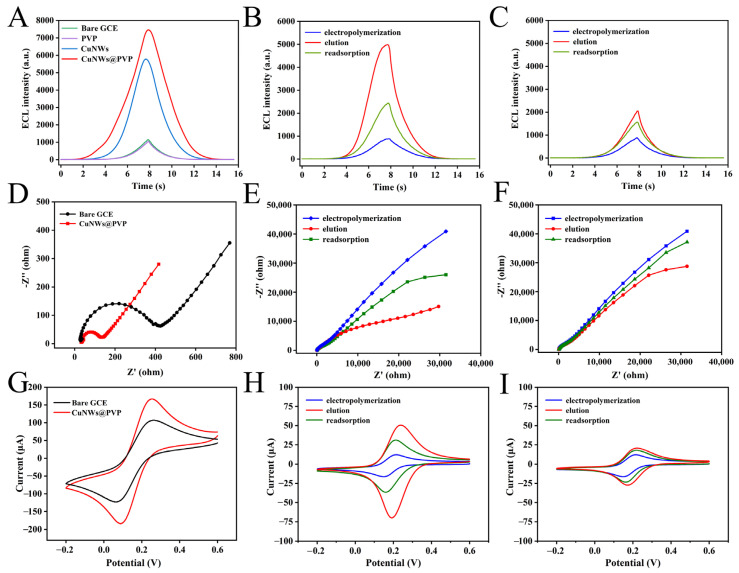
The ECL spectra (**A**), EIS curves (**D**), and CV curves (**G**) of different modified electrodes; the ECL spectra (**B**), EIS curves (**E**), and CV curves (**H**) of MIP/CuNWs@PVP/GCE; the ECL spectra (**C**), EIS curves (**F**), and CV curves (**I**) of NIP/CuNWs@PVP/GCE.

The ECL behavior of the modified electrode at various stages was meticulously studied and analyzed. As depicted in Figure 3A, in a PBS solution containing 0.1 M K_2_S_2_O_8_ as the co-reactant, both the bare/GCE and the PVP/GCE exhibited negligible ECL signals. Upon the introduction of CuNWs into the ECL system as a luminescent probe, its exceptional conductivity accelerated the electron transfer rate, yielding good NIR ECL signals. When the macromolecular surfactant PVP with a long-chain structure adhered to the CuNWs surface, it created a spatial hindrance effect around the CuNWs, effectively preventing their mutual proximity and aggregation. The structurally stable PVP, as a protective film of CuNWs, further enhanced their dispersion stability. Consequently, the self-enhanced functionalized CuNWs@PVP exhibited remarkable NIR ECL emission. EIS analysis holds a key position in assessing the electrochemical properties of materials, with the semicircle diameter in the high-frequency region serving as an indicator of charge transfer resistance (Rct), where a smaller diameter corresponds to a lower resistance value and signifies a more efficient kinetic process within the electrochemical reaction. Meanwhile, the peak current on the CV curves serves as another significant metric for gauging the electron transfer rate. The EIS and CV curves presented in Figure 3D,G also demonstrated that the composite luminescent CuNWs@PVP exhibited excellent electronic conductivity. The ECL emission mechanism of CuNWs@PVP/GCE was as follows:

CuNWs@PVP + e^−^ *→* CuNWs@PVP^−•^ (1)

S_2_O_8_^2−^ + e^−^ *→* SO_4_^−•^ + SO_4_^2−^ (2)

CuNWs@PVP^−•^ + SO_4_^−•^ *→* CuNWs@PVP* + SO_4_^2−^ (3)

CuNWs@PVP* *→* CuNWs@PVP + *hv* (4)

*—excited state.

### 3.4. MIP/CuNWs@PVP/GCE Response Behavior to CIP

When the MIP membrane was electropolymerized onto the surface of CuNWs@PVP/GCE (Figure 2D), the dense and non-conducting MIP membrane not only obstructed the transport channels between active ions (S_2_O_8_^2−^, SO_4_^−•^, etc.), but also impeded the electron transfer rate, resulting in drastically decreased ECL signals (Figure 3B), and CV values (Figure 3H), and a notable increase in EIS values (Figure 3E). The imprinted pores were formed following the elution of CIP, and the tunnel-shaped pores provided a pathway for the transfer of electrons and reactive ions, the ECL signals and CV values increased (Figure 3B,H), and the EIS values decreased (Figure 3E). Upon re-immersion of the eluted MIP/CuNWs@PVP/GCE into a CIP-containing environment, the capture of CIP molecules by the imprinted sites led to cavity blockage and subsequent interruption of electron and reactive ion channels, resulting in decreased ECL signals and CV values, and increased EIS values (Figure 3B,H,E). Given that NIP/CuNWs@PVP/GCE was absent of CIP to create imprinted holes, there was no significant change in ECL signals, EIS values, and peak current values before and after elution, as well as during the readsorption process (Figure 3C,F,I).

### 3.5. Condition Optimization of MIP/CuNWs@PVP/GCE

**Figure 4:**
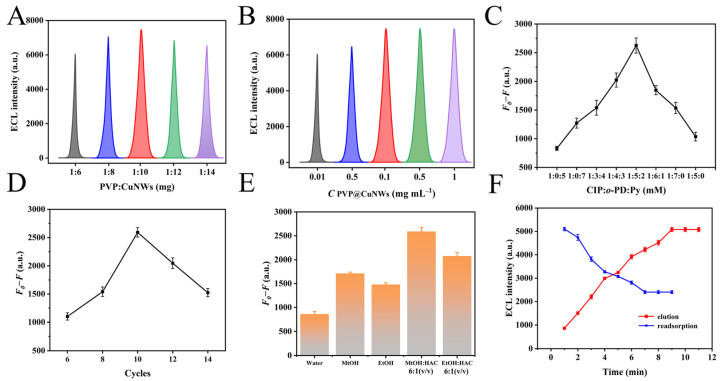
Optimization of ratio between PVP and CuNWs (**A**); the concentration optimization for CuNWs@PVP (**B**); optimization of the ratio of template molecules (CIP) to bifunctional monomers (o-PD: Py) (**C**); optimization of the number of cycles of MIP electropolymerization (**D**); optimization of eluent types (**E**); optimization of elution time and readsorption time (**F**).

The stabilizer and dispersant PVP were effectively coated on the CuNWs surface in appropriate proportions to prevent agglomeration of CuNWs. According to the results presented in Figure 4A, satisfactory ECL signals were achieved when the ratio of PVP to CuNWs was 1:10. With CuNWs@PVP as the main ECL emitter, the intensity of the ECL on the electrode surface augmented as the concentration of CuNWs@PVP increased, and reaching a stable level at a concentration of 0.1 mg mL^−1^ (Figure 4B).

The ratio of template molecules (CIP) to bifunctional monomers (*o*-PD:Py) exerted a direct impact on the structure of the MIP and the abundance of imprinted pores. According to the optimization results depicted in Figure 4C, the MIP formed with CIP and a single functional monomer demonstrated a commendable ECL quenching effect (*F*_0_ − *F*, *F*_0_: the ECL signals after elution, *F*: the ECL signals after readsorption). A more pronounced quenching effect was observed in bifunctional monomers MIECLS. This was due to the bifunctional monomers with more affinity sites leading to the retention of a greater number of imprinted pores following elution. Furthermore, mitigating the collapse of the imprinted pores caused by repeated elution during the detection process. The MIECLS exhibited a heightened response sensitivity to CIP when the ratio of CIP, *o*-PD, and Py was adjusted to 1:5:2. The thickness of the MIP layer is directly influenced by the number of electropolymerization cycles, which subsequently has an effect on the recognition capabilities of MIECLS. A lower number of electropolymerization cycles led to a thinner MIP layer, resulting in a decreased number of imprinted sites after elution. As the number of cycles gradually increased, more CIP molecules participated in the MIP construction process. However, an excessively high number of cycles caused the MIP membrane to be overly thick, hindering the effective elution of CIP molecules. As illustrated in Figure 4D, optimal results were achieved with 10 cycles. The selection of the eluent is pivotal for the elution of CIP, as it determines whether CIP can be eluted swiftly. Figure 4E demonstrated that the optimal effect was when the ratio of methanol to acetic acid was 6:1 (*v*/*v*). Figure 4F optimized the elution and adsorption time, and the MIP/CuNWs@PVP/GCE precursors were placed in an elution solution with methanol and acetic acid of 6:1 (*v*/*v*), the ECL intensity gradually recovered, and when the elution time reached 9 min, it reached equilibrium. It was observed that the ECL signals were stable when the eluted MIP/CuNWs@PVP/GCE was reabsorbed in CIP solution for 7 min.

### 3.6. Analysis of MIECLS Application Performance

**Figure 5:**
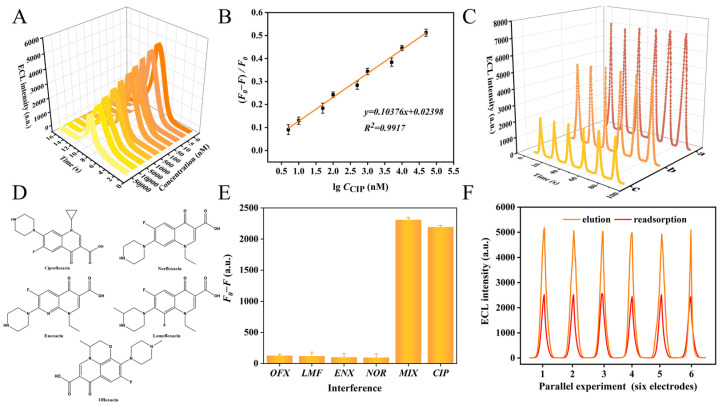
ECL responses of MIP/CuNWs@PVP/GCE under different CIP concentrations (**A**); standard curve for detecting CIP (**B**); stability test of CuNWs@PVP/GCE (a), and elution (b) and readsorption (c) of MIP/CuNWs@PVP/GCE (**C**); molecular formulas of CIP and its interfering substances (**D**); selective test (OFX: ofloxacin, LMF: lomefloxacin, ENX: enoxacin, and NOR: norfloxacin) of MIECLS (**E**); reproducibility test of MIECLS (**F**).

The precursor of MIP with CIP as template molecules was electropolymerized on the surface of CuNWs@PVP/GCE, and its insulating and spatial site-blocking effects cut off the contact paths between the self-enhanced luminescent probe CuNWs@PVP and the co-reactant K_2_S_2_O_8_, hindering the electron transfer and leading to the drop of ECL signals. When MIP/CuNWs@PVP/GCE was eluted by the eluent to leave behind imprinted pores with a spatial structure and three-dimensional size matching the CIP, this provided a transfer channel for electrons and reactive ions between CuNWs@PVP and K_2_S_2_O_8_, recovering the ECL signals. When the eluted MIP/CuNWs@PVP/GCE was immersed in an environment containing CIP, the imprinted pores would recapture CIP, causing a decrease in the ECL signals. Based on this mechanism, the relationship between the logarithm of CIP concentration and ECL intensity was utilized to construct a method for the selective detection of CIP. As depicted in Figure 5A, the ECL intensity of MIP/CuNWs@PVP/GCE decreased with the increase in CIP concentration (5.00 × 10^−9^ mol L^−1^–5.00 × 10^−5^ mol L^−1^). In this concentration range, the standard curve was plotted with *F*_0_ − *F*/*F*_0_ as the vertical coordinate and lg CCIP as the horizontal coordinate (Figure 5B), and the linear equation was *F*_0_ − *F*/*F*_0_ = 0.10376 lg C_CIP_ + 0.02398 (R^2^ = 0.9917) with a limit of detection (LOD) of 2.59 × 10^−9^ mol L^−1^.

### 3.7. Stability, Selectivity, Reproducibility, and Regeneration Analysis of MIECLS

The stability of the sensor indicates its capacity to maintain the output signal substantially constant over an extended period of usage. The stability of key parameters during the MIECLS construction process was demonstrated in Figure 5C. After six consecutive scans of the CuNWs@PVP/GCE, the relative standard deviation (RSD) was 1.37%, and the RSD values of MIP/CuNWs@PVP/GCE after elution and readsorption were 0.61% and 2.53%, respectively. These results indicated that the constructed MIECLS in this work had good stability.

The selectivity of MIECLS is essential to ensure a specific response to CIP (Figure 5E). NOR, ENX, LMF, and OFX, as structural analogs that may coexist with CIP, were selected as interferents (Figure 5D). Despite the fact that the concentration of these interferents (1.00 × 10^−4^ mol L^−1^) was ten times higher than that of CIP, MIECLS demonstrated the ability to accurately detect CIP in the presence of either a single interferent or a mixture of multiple interferents, indicating that the shape and size of the imprinted pores left behind after elution was highly matched with the molecular structure of CIP.

The reproducibility of the sensor is of great significance to ensure the accuracy and reliability of the measurement results. To assess the reproducibility of the MIECLS, six MIP/CuNWs@PVP/GCEs were fabricated employing the identical methodology depicted (Figure 5F). The ECL intensities of these electrodes were meticulously documented after elution and readsorption. Notably, the RSD of the quenching values among six electrodes amounted to 2.75%, demonstrating the satisfactory reproducibility of the MIECLS.

The regeneration of MIECLS holds immense significance in terms of cost reduction, the enhancement of detection efficiency, and fostering environmental sustainability, serving as a significant factor in the advancement and application of modern sensing technology. As illustrated in Figure S1, following five cycles of elution and readsorption tests, the MIP/CuNWs@PVP/GCE exhibited an ECL intensity retention rate exceeding 95%, which highlighted the good regeneration of the MIECLS to detect CIP in complex matrices during practical application.

### 3.8. Assessment of MIECLS Performance in Actual Samples

The performance of constructed MIECLS was evaluated using pork and fish real samples, with concentrations of 20 μg kg^−1^, 30 μg kg^−1^, and 40 μg kg^−1^ being selected for conducting spiked recovery experiments. As indicated in Table 1, no CIP was detected in the blank sample, and the recovery rates of CIP range from 84.39% to 92.48%, conclusively demonstrating the capacity of the MIECLS to achieve precise CIP detection. Compared with other CIP detection methods (Table 2), the constructed MIECLS in this work presented a lower LOD and a broader range of CIP detection concentrations, which has significant advantages in the specificity and high sensitivity detection of CIP.

## 4. Conclusions

In this work, a functionalized PVP membrane was in situ attached to the CuNWs surface to prevent CuNWs deposition and agglomeration and realize the self-enhanced NIR ECL signals. MIP with bifunctional monomers not only overcame the problem of the limited number of imprinting cavities, but also significantly improved the affinity between the target substances and the functional monomers, thus greatly enhancing the detection sensitivity and accuracy of CIP. According to the above strategy, a bifunctional monomer molecularly imprinted sensor based on the emerging NIR ECL probe PVP@CuNWs has been successfully used for trace-targeted recognition of the harmful substance CIP in food. The experimental results demonstrated that the constructed sensor showed satisfactory selectivity, stability, reproducibility, and regeneration. We introduced a low-cost, environmentally friendly, and straightforward method for constructing a highly penetrative ECL sensing platform through the design of a copper-based NIR photoelectric network, and provided a new technological pathway in the field of food safety and environmental monitoring for the efficient detection of hazardous combing with the updated MIP.

## Figures and Tables

**Table 1:** Determination of CIP in real samples by the prepared MIECLS.

	Spiked (μg kg^−1^)	Found (μg kg^−1^)	Recovery (%) (Mean ± SD) (*n* = 3)
Pork	0	Nd ^a^	–– ^a^
	20	17.08	85.44 ± 1.32
	30	27.00	90.03 ± 2.15
	40	36.62	91.56 ± 1.89
Fish	0	Nd ^a^	–– ^a^
	20	16.88	84.39 ± 1.45
	30	27.74	92.48 ± 0.79
	40	36.44	91.09 ± 1.37

^a^ Not detectable.

**Table 2:** Comparison with other reported methods for detecting CIP.

Methods	Linear Range (M)	LOD (M)	Reference
SERS ^a^	1.00 × 10^−8^–1.00 × 10^−4^	5.20 × 10^−9^	[21]
Colorimetric	5.00 × 10^−5^–2.00 × 10^−4^	2.90 × 10^−8^	[22]
Electrochemiluminescence	5.00 × 10^−7^–2.00 × 10^−4^	5.00 × 10^−7^	[23]
Electrochemical	1.00 × 10^−5^–3.00 × 10^−3^	5.88 × 10^−7^	[24]
Electrochemical	1.00 × 10^−6^–3.00 × 10^−5^	1.08 × 10^−7^	[25]
Fluorescence	5.00 × 10^−6^–2.00 × 10^−4^	3.30 × 10^−6^	[26]
Fluorescence	8.00 × 10^−8^–5.00 × 10^−5^	1.80 × 10^−8^	[27]
HPLC-UV ^b^	1.50 × 10^−7^–2.40 × 10^−5^	3.00 × 10^−8^	[28]
HPLC-MS/MS ^c^	3.00 × 10^−6^–2.00 × 10^−4^	2.50 × 10^−7^	[29]
Electrochemiluminescence	5.00 × 10^−9^–5.00 × 10^−5^	2.59 × 10^−9^	This work

^a^ Surface Enhanced Raman Spectroscopy, ^b^ High Performance Liquid Chromatography (Ultraviolet Detector), ^c^ High Performance Liquid Chromatography-Tandem Mass Spectrometry.

## Data Availability

The original contributions presented in the study are included in the article and Supplementary Materials, further inquiries can be directed to the corresponding author.

## Supplementary Materials

The following supporting information can be downloaded at: https://www.mdpi.com/article/10.3390/foods14030538/s1, Figure S1. The regeneration test of MIECLS.
