# Supplementary material for: Self-Enhanced Near-Infrared Copper Nanoscale Electrochemiluminescence Probe for the Sensitive Detection of Ciprofloxacin in Foods
Source: Foods. 2025 Feb 6;14(3):538. doi: 10.3390/foods14030538 (PMC11816994; doi:10.3390/foods14030538)
Supplement: Supplementary file 1 [file foods-14-00538-s001.zip › foods-3435296-supplementary.pdf]

# Self-Enhanced Near-Infrared Copper Nanoscale Electrochemiluminescence Probe for the Sensitive Detection of Ciprofloxacin in Foods

**Jie Wu, Yuanjie Qin, Xiaoxin Mei, Lin Cai, Wen Hao and Guozhen Fang ***

State Key Laboratory of Food Nutrition and Safety, Tianjin University of Science and Technology, Tianjin 300457, China; 22845807@mail.tust.edu.cn (J.W.); qinyuanjie@mail.tust.edu.cn (Y.Q.); meixiaoxin@mail.tust.edu.cn (X.M.); cailin@mail.tust.edu.cn (L.C.); haowen@mail.tust.edu.cn (W.H.)

* Correspondence: fangguozhen@tust.edu.cn

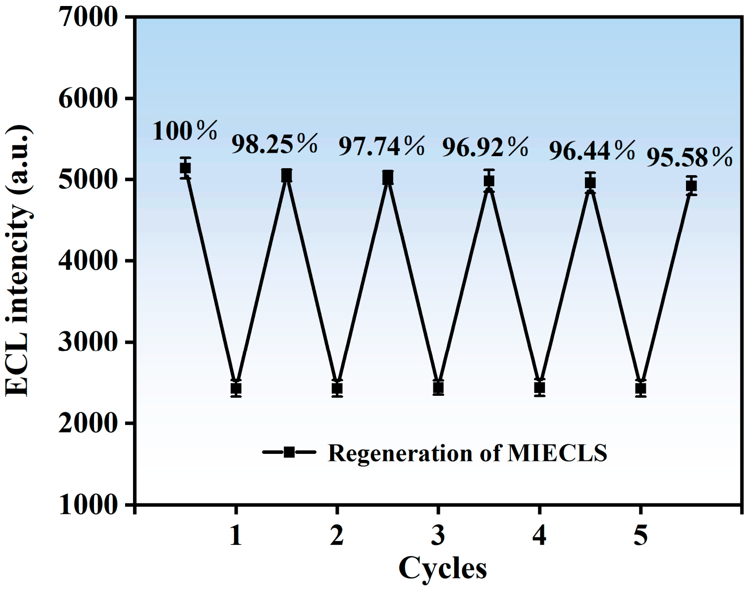


**Figure S1.** The regeneration test of MIECLS.
